# Translational repression controls temporal expression of the *Plasmodium berghei* LCCL protein complex^☆^

**DOI:** 10.1016/j.molbiopara.2013.04.006

**Published:** 2013-05

**Authors:** Sadia Saeed, Victoria Carter, Annie Z. Tremp, Johannes T. Dessens

**Affiliations:** Department of Pathogen Molecular Biology, Faculty of Infectious and Tropical Diseases, London School of Hygiene & Tropical Medicine, London WC1E 7HT, United Kingdom

**Keywords:** Malaria transmission, Transgenic parasite, Translational repression, Crystalloid

## Abstract

*Plasmodium* LCCL proteins comprise a family of six proteins that function as a protein complex and have essential roles in sporozoite transmission. In *Plasmodium berghei*, family members *Pb*LAP1, *Pb*LAP2 and *Pb*LAP3 have been shown to be expressed in gametocytes and, following gametogenesis and fertilization, to be targeted to distinctive multivesicular organelles termed crystalloids that form in the ookinete. Here, we show by GFP-tagging that *Pb*LAP4, *Pb*LAP5 and *Pb*LAP6, like their family members, are associated with the crystalloids. However, in contrast to their family members, protein expression of *Pb*LAP4, *Pb*LAP5 and *Pb*LAP6 was not detected in gametocytes, even though transcription of the corresponding genes is most prominent in the sexual blood stage parasites. These results suggest that translational repression controls expression of the LCCL protein repertoire and, consequently, the temporal function of the protein complex during *P. berghei* development in the mosquito.

## Highlights

• We have GFP-tagged the LCCL proteins *Pb*LAP4, *Pb*LAP5 and *Pb*LAP6 in *Plasmodium berghei*.
• *Pb*LAP4, *Pb*LAP5 and *Pb*LAP6 associate with the crystalloid organelle in ookinetes.
• Translational repression controls expression of the LCCL protein repertoire in gametocytes.

## Graphical abstract

**Figure:**
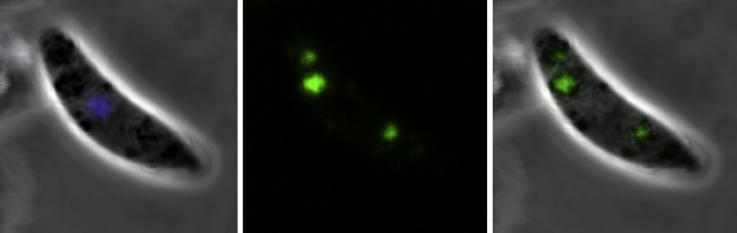

Crystalloids are unique parasite structures implicated in malaria transmission by virtue of their restricted presence in ookinetes and young oocyst stages that develop in the mosquito vector (reviewed in [1]). Crystalloids appear in transmission electron microscopy (TEM) as large clusters of small spherical particles. *Plasmodium falciparum* crystalloids are virtually indistinguishable from those of *P. berghei* and high resolution electron micrographs show their subunit particles to be individually bound by a lipid bilayer [2]. Hence, *Plasmodium* crystalloids appear to be multivesicular organelles rather than particulate cytoplasmic inclusions. To date, three parasite proteins have been found associated with the crystalloids: *Pb*LAP1 (also known as *Pb*SR), *Pb*LAP2 and *Pb*LAP3, all members of a gametocyte-expressed family of LCCL-lectin adhesive-like domain proteins (LAPs) otherwise known as *Plasmodium* LCCL proteins or *P*CCp proteins [3–7]. This link is based not only on the presence of these three *Pb*LAP family members in the crystalloids, but also by the failure of *Pb*LAP1 knockout and deletion mutants to form crystalloids [4,5]. The LAP family is composed of six highly conserved and structurally related proteins with a modular architecture consisting of multiple domains implicated in protein, lipid and carbohydrate binding. Family members are both typified by, and named after, the LCCL domain that was initially identified in the horse shoe crab *Limulus* clotting factor C, the cochlear protein Coch-5b2, and the late gestation lung protein Lgl1 [8]. The LCCL domain is present either in single or multiple copies in all but one family member. LCCL proteins also possess a canonical amino-terminal endoplasmic reticulum (ER) signal peptide and are translocated into the ER [4].

With the exception of *Pb*LAP3, all *Pb*LAP family members have been studied by targeted gene disruption and show very similar loss-of-function phenotypes epitomized by a failure to form sporozoites in the oocysts (Table 1). This is not a foolproof genetic block as *Pb*LAP knockout parasites occasionally produce sporulating oocysts, however sporozoite transmission has not been achieved with any of the *Pb*LAP null mutant lines [4,6,9,10]. The apparent oocyst/sporozoite-associated function of the LCCL proteins is poorly compatible with their reported expression in gametocytes as these life cycle stages are several days and developmental transitions apart, and it has been proposed that the crystalloids provide a protein trafficking mechanism to deliver the LCCL proteins, and possibly other molecules, from the gametocyte to the oocyst [4,11]. The gametocyte-specific expression profiles of all the *Pb*LAPs as ascertained by fluorescent protein tagging, GFP reporter studies and RT-PCR, combined with their very similar loss-of-function phenotypes, points to a functional co-dependence and indicates that these molecules are involved in a similar cell biological process and operate in concert, probably as a protein complex [1]. This is further supported by observations that simultaneous knockout of two family members gives the same phenotype as the single knockouts, showing a lack of functional redundancy [12]. Indeed, molecular interactions between LCCL protein family members have been shown in *P. falciparum* using co-immunoprecipitation experiments [13]. Moreover, a mechanism promoting interaction between family members based on conformational interdependence was recently reported [14].

In this study we investigated whether the LCCL proteins *Pb*LAP4 (PBANKA_131950), *Pb*LAP5 (PBANKA_131530) and *Pb*LAP6 (PBANKA_041760) displayed similar expression profiles as their family members, as well as an association with the crystalloids. Our strategy to investigate this was to generate genetically modified parasite lines in which the native proteins were fused at their carboxy-terminus with enhanced GFP, a strategy used successfully for *Pb*LAP1, *Pb*LAP2 and *Pb*LAP3 [4,5]. This allows *Pb*LAP::GFP fusions to be expressed from the endogenous promoter, but with a 3′UTR derived from the *pbdhfr* gene [4,5]. To achieve GFP-tagging of *Pb*LAP6 we adopted a strategy of single crossover homologous recombination. A ca. 1.9 kb fragment of *pblap6* corresponding to the 3′-part of the coding sequence was PCR amplified from genomic DNA with primers P1 (ACGAAGTTATCAGTCGACAGCCCCAGTTCAGACATAAAC) and P2 (ATGAGGGCCCCTAAGCTTTCTTTATGAGGAATAAATAAAATGTTTTTAAAC) (Fig. 1A) and introduced into *Sal*I/*Hin*dIII-digested pDNR-EGFP [4] via in-fusion cloning (Takara Biotech) to give plasmid pDNR-PbLAP6/EGFP. The *pblap6/egfp* specific sequence was then transferred to pLP-hDHFR [5] via cre-*lox*p recombination to give plasmid pLP-PbLAP6/EGFP (Fig. 1A). The same strategy was used to tag *Pb*LAP4 at its carboxy terminus. A ca. 3 kb fragment of *pblap4* corresponding to the 3′-part of the coding sequence was PCR amplified from genomic DNA with primers P3 (ACGAAGTTATCAGTCGACAAGATGTCGAAAATATTTGTGCAT) and P4 (ATGAGGGCCCCTAAGCTTTCACATTCTGATATACACTGATTTATCA) and introduced into *Sal*I/*Hin*dIII-digested pLP-PbLAP6/EGFP to give pLP-PbLAP4/EGFP (Fig. 1A). To achieve GFP-tagging of *Pb*LAP5 we used a strategy of double crossover homologous recombination. The entire *pblap5* coding sequence plus ca. 0.6 kb of upstream sequence was PCR amplified from genomic DNA with primers P5 (ACGAAGTTATCAGTCGAAGCTTCATACTGTTATATATTGCACATATAGCC) and P6 (ATGAGGGCCCCTAAGCTATTGTGGAGAAATATAATTTGTATAGATTG) (Fig. 1A) and cloned into *Sal*I/*Hin*dIII-digested pDNR-EGFP to give plasmid pDNR-PbLAP5/EGFP. The 3′UTR of *pblap5* was amplified with primers P7 (CCTTCAATTTCGACATAGAGGCATTTGACAAACAAAC) and P8 (GCGGCCGCTCTAGCATAATGTTTTATTTTTTCCATTTTCAGC) (Fig. 1A) and the resulting ca. 0.7 kb fragment cloned into *Nde*I-digested pLP-hDHFR by in-fusion cloning to give plasmid pLP-hDHFR/PbLAP5. The *pblap5/egfp*-specific sequence from pDNR-PbLAP5/EGFP was transferred to pLP-hDHFR/PbLAP5 by cre/*lox*p recombination to give the final construct pLP-PbLAP5/EGFP (Fig. 1A). Plasmid pLP-PbLAP5/EGFP was linearized with *Hin*dIII and *Sac*II to remove the vector backbone prior to transfection, whereas plasmids pLP-PbLAP6/EGFP and pLP-PbLAP4/EGFP were linearized with *Xho*I and *Pac*I, respectively. After transfection pyrimethamine-resistant parasites were selected and cloned, as described [15], to give parasite lines *Pb*LAP4/GFP, *Pb*LAP5/GFP and *Pb*LAP6/GFP, respectively.

Diagnostic PCR on genomic DNA extracted from clonal lines of the transgenic parasites was used to confirm the correct integration of the modified alleles into their target loci, as well as the absence of the wildtype alleles. Primers P9 (CACAATTGGTATAACACCG) and P10 (GTGCCCATTAACATCACC) (Fig. 1A) amplified a unique ca. 2.0 kb fragment from parasite line *Pb*LAP6/GFP (Fig. 1B), confirming correct integration of the *gfp* sequence downstream of the *pblap6* allele. Conversely, primers P9 and P11 (CCTTTTATATTTTGTACCCATTTAATCG) (Fig. 1A) amplified a predicted fragment of ca. 2.0 kb from WT parasites, but not from *Pb*LAP6/GFP parasites (Fig. 1B), demonstrating the absence of the wildtype *pblap6* allele in the transgenic lines. Similarly, diagnostic PCR using primers P12 (GCCTAGTTCTCCTTCTGG) and P10 (Fig. 1A) amplified a unique ca. 3.3 kb fragment from parasite line *Pb*LAP4/GFP (Fig. 1B), confirming correct integration of the *gfp* sequence downstream of the *pblap4* allele. Conversely, primers P12 and P13 (TTTGATAGCACTCTTTCAAATGC) (Fig. 1A) amplified a predicted fragment of ca. 3.3 kb from WT parasites only (Fig. 1B). Diagnostic PCR using primers P14 (ACAAAGAATTCATGGTTGGTTCGCTAAACT) and P15 (CTCTTCCAATTGCTCATTTA) (Fig. 1A) amplified a unique ca. 1.6 kb fragment from parasite line *Pb*LAP5/EGFP (Fig. 1B), confirming correct integration of the *hdhfr* selectable marker gene cassette into the *pblap5* locus. Moreover, the absence of WT parasites was confirmed using primers P5 and P13 that amplified a ca. 4.5 kb fragment specific to the WT *pblap5* allele (Fig. 1B). Validated *Pb*LAP4/GFP, *Pb*LAP5/GFP and *Pb*LAP6/GFP clonal parasite lines exhibited normal asexual and sexual blood stage development.

GFP fluorescence was not detected in mature oocysts of parasite lines *Pb*LAP4/GFP, *Pb*LAP5/GFP and *Pb*LAP6/GFP, consistent with the demonstrated lack of discernible transcription of these *pblap* genes in oocysts and sporozoites by GFP reporter and RT-PCR studies [12]. To assess *Pb*LAP protein expression and localization in ookinetes, ookinete cultures were set up from gametocytemic mouse blood. Mature ookinetes of parasite lines *Pb*LAP4/GFP, *Pb*LAP5/GFP and *Pb*LAP6/GFP exhibited GFP-based fluorescence that typically distributed to 2–3 regions visibly associated with clusters of malaria pigment, characteristic of crystalloid targeting (Fig. 2A). This localization pattern is very similar to that observed for *Pb*LAP1, *Pb*LAP2 and *Pb*LAP3 [4,5] indicating that – like their family members – *Pb*LAP4, *Pb*LAP5 and *Pb*LAP6 associate with the crystalloids. This adds further strong support to the concept that all the *Plasmodium* LCCL proteins are involved in a common molecular process and act as a protein complex, consistent with their common loss-of-function phenotypes and lack of functional redundancy. Furthermore, the targeting of *Pb*LAP5 to crystalloids demonstrates that the LCCL domain is not required to be present in individual family members in order for the protein to be sorted to this organelle. This argues against the concept that the LCCL domain constitutes an organellar-targeting signal for crystalloids.

We previously showed by GFP tagging that *Pb*LAP1, *Pb*LAP2 and *Pb*LAP3 are first expressed as protein in gametocytes [4,5]. It was therefore surprising that we could not observe green fluorescence in gametocytes of parasite lines *Pb*LAP4/GFP, *Pb*LAP5/GFP and *Pb*LAP6/GFP (Fig. 2A). To shed more light on this we investigated gene transcription of *pblap4*, *pblap5* and *pblap6* by total RNA extraction from wildtype *P. berghei* ANKA parasites followed by reverse transcription and PCR. For each gene tested we used primer pairs that flanked an intron in order to distinguish between amplification from cDNA or contaminating gDNA (Fig. 1A). Primers P16 (GCACTTTCTTTACGTGAATGGAG) and P17 (GCCAACTACTACGCCCATC) amplified a *pblap4* cDNA-specific predicted product of ca. 750 bp in samples enriched for gametocytes or ookinetes. A ca. 990 bp predicted product was amplified from gDNA owing to the presence of an intron situated in between the primer annealing sites (Fig. 2B). By contrast, *pblap*-specific cDNA was not amplified from asexual blood stages (derived from *P. berghei* ANKA clone 2.33 which does not produce gametocytes), indicating that the product in the gametocyte sample was not amplified from cDNA from contaminating asexual blood stages (Fig. 2B). Similarly, primers P18 (GATACATAAATGCTACAGTGAGAATTATGAC) and P19 (CCCATCGAACAGAAAAATGC) amplified a *pblap5* cDNA-specific predicted product of ca. 750 bp from sexual but not asexual blood stages (Fig. 2B). The same primers gave rise to a ca. 990 bp predicted product from gDNA due to an intron between the primer annealing sites (Fig. 2B). Primers P20 (CGCATGTATGTGTGAATGTAGC) and P21 (ACATTAATGCACCATTCCCAT) amplified a *pblap6* cDNA-specific predicted amplicon of ca. 670 bp from gametocytes. The amplification product with this primer pair from gDNA was ca. 980 bp, again because of an intron situated between the primer annealing sites (Fig. 2B). The *pbtub1* (encoding tubulin 1) cDNA-specific primers TUB1cDNA-F (TAGGACAGGCTGGTATCCAAG) and TUB1cDNA-R (CTTGTGGTGATGGCCAGC) amplified a predicted cDNA product of ca. 500 bp (ca. 1200 bp from gDNA) confirming the presence of cDNA (Fig. 2B). Because gametocytes contaminate the ookinete sample, but not the other way around, these results indicate that mRNA corresponding to *pblap4*, *pblap5* and *pblap6* is in fact most abundant in the sexual blood stages, consistent with other mRNA expression studies of these three genes [12]. This inverse relationship between mRNA and protein expression in gametocytes and ookinetes fits well with a scenario of translational repression (TR). TR is a process of translational silencing of mRNA that in *P. berghei* is specific to female gametocytes and involved in development of the parasite post-fertilization [16]. Indeed *pblap4*, *pblap5* and *pblap6* are predicted to be subject of TR in studies using DOZI (development of zygote inhibited) null mutants, while *pblap1*, *pblap2* and *pblap3* are not [16]. This is fully consistent with our observations reported here and in other studies [4,5]. In further support of TR, neither *Pb*LAP4, *Pb*LAP5 and *Pb*LAP6 were detected in the female gametocyte proteome, as opposed to *Pb*LAP1, *Pb*LAP2 and *Pb*LAP3 [17].

The similar loss-of function phenotypes of the *pblap* family members strongly imply that a complete *Pb*LAP repertoire is needed to form a functional LCCL protein complex and allow normal sporozoite transmission. It is tempting to speculate that a functional LCCL protein complex would also be necessary for any gametocyte-specific function of these proteins. The TR of *pblap4*, *pblap5* and *pblap6* identified in this study reveals a potential mechanism to reduce the gametocyte-specific expression of select *Pb*LAP family members and in doing so control the amount of functional LCCL protein complex that is present in gametocytes. Within the LCCL protein family *Pb*LAP2 and *Pb*LAP4 are structural paralogues, as are *Pb*LAP3 and *Pb*LAP5 [1]. It has been poorly understood why the parasite encodes such similar proteins from distinct genes. The notion that of each pair of the *Pb*LAP paralogues only one is translationally repressed points to a biological need for their differential expression, which may have been the reason behind their ancestral gene duplication. The situation in *P. berghei* appears to be different from that in *P. falciparum* where all the LCCL protein family members have been shown to be expressed as proteins during gametocytogenesis and to form a ‘complete’ multi-protein complex in mature gametocytes [13,18]. Thus, TR of the LCCL proteins in *P. falciparum* appears to be either absent, or less effective than that of its orthologues in *P. berghei*. The subcellular distribution of the LCCL proteins in *P. falciparum* gametocytes includes a vesicular secretion to the parasitophorous vacuole, resulting in exposure of the protein complex to the extracellular environment upon gametogenesis, to which various putative functions have been attributed [7,13,18–20]. Notably, a similar secretion of *Pb*LAPs to the parasitophorous vacuole of *P. berghei* gametocytes is not apparent, although it cannot be ruled out that this occurs at low levels that are difficult to detect [4,5]. Besides considerable dissimilarities in gametocytogenesis, the TR of select *Pb*LAP family members could be a reason behind these species-specific differences.

## Figures and Tables

**Fig. 1:**
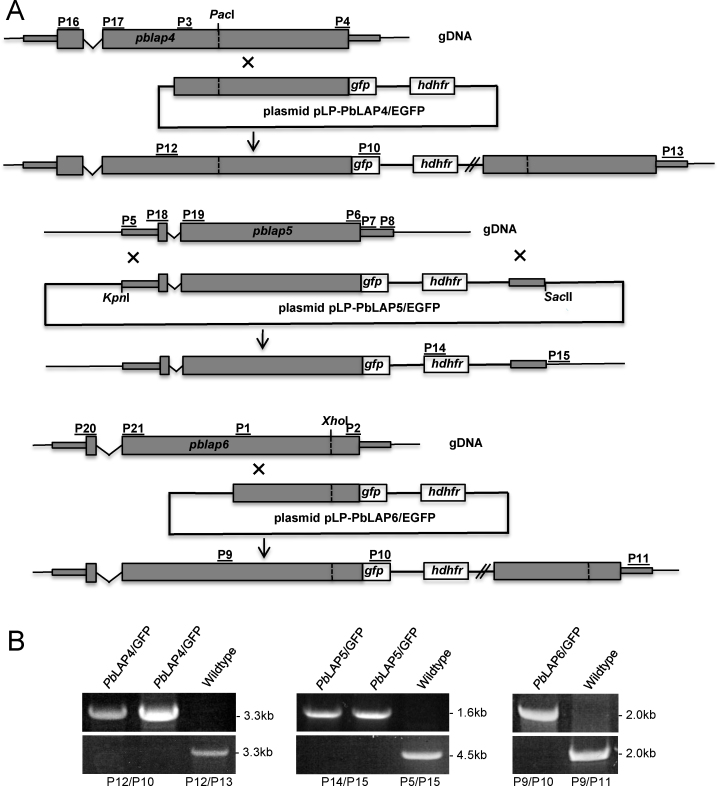
Generation and molecular analysis of genetically modified *pblap* parasite lines. (A) Targeting strategy for the GFP tagging of *Pb*LAP4, *Pb*LAP5 and *Pb*LAP6 via crossover homologous recombination. The *pblap* genes are indicated with coding sequence (wide bars) and untranslated regions (narrow bars). Also indicated are the enhanced GFP module (*gfp*); the hDHFR selectable marker gene cassette (*hdhfr*); introns (v-shaped line); key restriction sites (*Pac*I, *Xho*I, *Kpn*I, *Sac*II); and primers used for PCR amplification (P1-P21). (B) PCR diagnostic for the presence of modified GFP-tagged *pblap* alleles (top panels) and the absence of wildtype *pblap* alleles (bottom panels) from clonal parasite populations of *Pb*LAP4/GFP (left panel), *Pb*LAP5/GFP (middle panel) and *Pb*LAP6/GFP (right panel). Wildtype parasites are included as negative and positive controls, respectively. Approximate sizes (in kb) of PCR products are indicated.

**Fig. 2:**
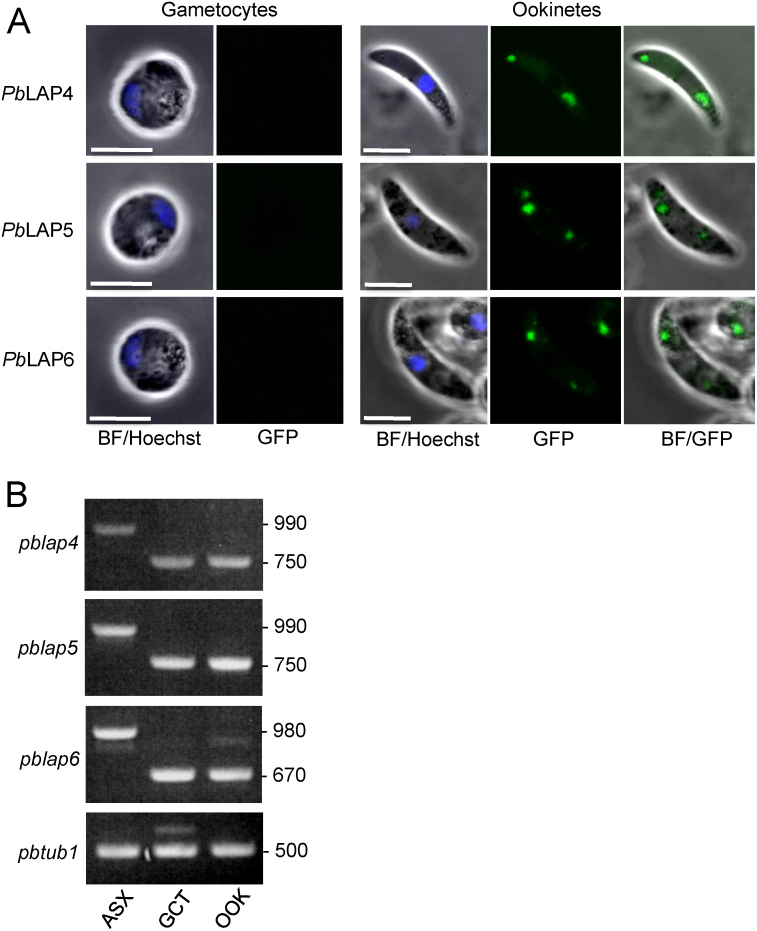
Gene expression and subcellular distribution of *pblap* gene products. (A) Confocal bright field and GFP images of gametocytes and ookinetes of parasite lines *Pb*LAP4/GFP, *Pb*LAP5/GFP and *Pb*LAP6/GFP. Both a longitudinal and transverse cross section of *Pb*LAP6/GFP ookinetes are shown. Hoechst DNA stain (blue) marks position of nucleus. Scale bar = 5 μm. (B) PCR on genomic DNA (gDNA) and cDNA from parasite samples enriched for asexual blood stages (ASX), gametocytes (GCT) and ookinetes (OOK), using primers specific for *pblap4* (P16/P17), *pblap5* (P18/P19), *pblap6* (P20/P21) and the control gene *pbtub1* (encoding tubulin 1). The relative positions of these primers are indicated in Fig. 1A. (For interpretation of the references to color in this figure legend, the reader is referred to the web version of the article.)

**Table 1:** Loss-of-function phenotypes of five LCCL protein family members of *Plasmodium berghei* in *Anopheles stephensi* mosquitoes.

PBANKA_000000^a^	103520	130070	131950	131530	041760
Name of gene product	*Pb*LAP1	*Pb*LAP2	*Pb*LAP4	*Pb*LAP5	*Pb*LAP6
Alternative name(s)	*Pb*CCp3 *Pb*SR	*Pb*CCp1	*Pb*CCp2	*Pb*PNFA	*Pb*CCp4
References	[4,6]	[10]	[10]	[9]	[10]

Crystalloid formation	Absent	n/a^b^	n/a	n/a	n/a
Gametogenesis	Normal				
Ookinete development	Normal				
Oocyst transition	Normal				
Sporogenesis^c^	Highly reduced				
Transmission	Not achieved				

a Loss-of-function phenotype of PBANKA_020450 (*Pb*LAP3) has not been published.

b Not assessed.

c Proportion of sporulating oocysts, rather than the level of sporulation per oocyst.

## References

[1] Dessens J.T., Saeed S., Tremp A.Z., Carter V. (2011). Malaria crystalloids: specialized structures for parasite transmission?. Trends in Parasitology.

[2] Meis J.F., Ponnudurai T. (1987). Ultrastructural studies on the interaction of *Plasmodium falciparum* ookinetes with the midgut epithelium of *Anopheles stephensi* mosquitoes. Parasitology Research.

[3] Trueman H.E., Raine J.D., Florens L., Dessens J.T., Mendoza J., Johnson J. (2004). Functional characterization of an LCCL-lectin domain containing protein family in *Plasmodium berghei*. Journal of Parasitology.

[4] Carter V., Shimizu S., Arai M., Dessens J.T. (2008). PbSR is synthesized in macrogametocytes and involved in formation of the malaria crystalloids. Molecular Microbiology.

[5] Saeed S., Carter V., Tremp A.Z., Dessens J.T. (2010). *Plasmodium berghei* crystalloids contain multiple LCCL proteins. Molecular and Biochemical Parasitology.

[6] Claudianos C., Dessens J.T., Trueman H.E., Arai M., Mendoza J., Butcher G.A. (2002). A malaria scavenger receptor-like protein essential for parasite development. Molecular Microbiology.

[7] Pradel G., Hayton K., Aravind L., Iyer L.M., Abrahamsen M.S., Bonawitz A. (2004). A multidomain adhesion protein family expressed in *Plasmodium falciparum* is essential for transmission to the mosquito. Journal of Experimental Medicine.

[8] Trexler M., Banyai L., Patthy L. (2000). The LCCL module. European Journal of Biochemistry.

[9] Ecker A., Bushell E.S., Tewari R., Sinden R.E. (2008). Reverse genetics screen identifies six proteins important for malaria development in the mosquito. Molecular Microbiology.

[10] Raine J.D., Ecker A., Mendoza J., Tewari R., Stanway R.R., Sinden R.E. (2007). Female inheritance of malarial *lap* genes is essential for mosquito transmission. PLoS Pathogens.

[11] Garnham P.C., Bird R.G., Baker J.R., Desser S.S., el-Nahal H.M. (1969). Electron microscope studies on motile stages of malaria parasites. VI. The ookinete of *Plasmodium berghei* yoelii and its transformation into the early oocyst. Transactions of the Royal Society of Tropical Medicine and Hygiene.

[12] Lavazec C., Moreira C.K., Mair G.R., Waters A.P., Janse C.J., Templeton T.J. (2009). Analysis of mutant *Plasmodium berghei* parasites lacking expression of multiple PbCCp genes. Molecular and Biochemical Parasitology.

[13] Simon N., Scholz S.M., Moreira C.K., Templeton T.J., Kuehn A., Dude M.A. (2009). Sexual stage adhesion proteins form multi-protein complexes in the malaria parasite *Plasmodium falciparum*. Journal of Biological Chemistry.

[14] Saeed S., Tremp A.Z., Dessens J.T. (2012). Conformational co-dependence between *Plasmodium berghei* LCCL proteins promotes complex formation and stability. Molecular and Biochemical Parasitology.

[15] Janse C.J., Ramesar J., Waters A.P. (2006). High-efficiency transfection and drug selection of genetically transformed blood stages of the rodent malaria parasite *Plasmodium berghei*. Nature Protocols.

[16] Mair G.R., Braks J.A., Garver L.S., Wiegant J.C., Hall N., Dirks R.W. (2006). Regulation of sexual development of Plasmodium by translational repression. Science.

[17] Khan S.M., Franke-Fayard B., Mair G.R., Lasonder E., Janse C.J., Mann M. (2005). Proteome analysis of separated male and female gametocytes reveals novel sex-specific Plasmodium biology. Cell.

[18] Scholz S.M., Simon N., Lavazec C., Dude M.A., Templeton T.J., Pradel G. (2008). PfCCp proteins of *Plasmodium falciparum*: gametocyte-specific expression and role in complement-mediated inhibition of exflagellation. International Journal for Parasitology.

[19] Delrieu I., Waller C.C., Mota M.M., Grainger M., Langhorne J., Holder A.A. (2002). PSLAP: a protein with multiple adhesive motifs, is expressed in *Plasmodium falciparum* gametocytes. Molecular and Biochemical Parasitology.

[20] Pradel G., Wagner C., Mejia C., Templeton T.J. (2006). *Plasmodium falciparum*: co-dependent expression and co-localization of the PfCCp multi-adhesion domain proteins. Experimental Parasitology.

